# Retraction: Effects of naturally occurring six- and twelve-nucleotide inserts on newcastle disease virus replication and pathogenesis

**DOI:** 10.1371/journal.pone.0244044

**Published:** 2020-12-14

**Authors:** The PLOS ONE Editors

After this article [1] was published, concerns were raised about results reported in Figs 3, 4, and 6, and in Table 1.

Specifically:

- The rBan panel in Fig 3d of [1] appears similar to the ‘wt Ban/010’ panel in the top row of Fig 1c of [2], which has been retracted [3]. The authors provided image data to support the experiment reported in [1] and noted that they cannot comment on why the image was duplicated in [2].
- In Fig 4a, there appear to be vertical discontinuities in the background after lanes 1, 2, and 3 in each of the two blot panels. The authors commented that due to the strong reactivity of the N antibody, the samples were run in non-adjacent SDS-PAGE lanes. The original data needed to clarify this issue are not available, although the authors offered replication data to support this result.
- The results reported in Fig 6 raised concerns about animal welfare and ethics considerations in the in vivo study. The article and information provided post-publication differ in descriptions of experimental endpoints and how animal clinical symptoms were scored during the in vivo experiments. Furthermore, the descriptions reported in the article for clinical scores of 2 or 3 (paralysis/twitching/wing drop, prostration) seem to align with humane endpoint criteria outlined in the study protocol (inactive, not responding to stimuli”, “Incoordination, including loss of ability to walk and stand”), according to documentation provided to the journal post-publication. In response to this issue, the author noted that all animal experiments were performed according to the IACUC protocol.
- In Table 1, all reported SEM (standard error of the mean) values end in either 0 or 5. The authors apologized, noted that errors were made in reporting SEM values in this Table, and provided raw data to support these results. However, the information provided post-publication did not fully clarify questions raised about the published results.

In light of these concerns, the *PLOS ONE* Editors retract this article.

AP, SKS, and PLC did not agree with retraction. AP and PLC apologize for the issues with the published article. SKS and PLC stand by the article’s findings. The other authors either could not be reached or did not respond directly.
